# Differential NF2 Gene Status in Sporadic Vestibular Schwannomas and its Prognostic Impact on Tumour Growth Patterns

**DOI:** 10.1038/s41598-017-05769-0

**Published:** 2017-07-14

**Authors:** Hongsai Chen, Lu Xue, Hantao Wang, Zhaoyan Wang, Hao Wu

**Affiliations:** 1 Department of Otolaryngology Head & Neck Surgery, The Ninth People’s Hospital, Shanghai Jiao Tong University School of Medicine, Shanghai, China; 2 Department of Otolaryngology Head and Neck Surgery, Xinhua Hospital, Shanghai Jiao Tong University School of Medicine, Shanghai, China; 3 Ear Institute, School of Medicine, Shanghai Jiao Tong University, Shanghai, China; 4 Shanghai Key Laboratory of Translational Medicine on Ear and Nose Diseases, Shanghai, China

## Abstract

The great majority of sporadic vestibular schwannomas (VSs) are due to the inactivation of the *NF2* gene. In this study, we found age-dependent differences in the clinical parameters of sporadic VSs. Young patients were characterized by progressive tumour behaviours, including earlier onset of initial symptoms, shorter symptom duration and larger tumour size. An increased rate of “two-hits” of both *NF2* alleles, usually by mutation and allelic loss, was observed in young cases compared to older, and this correlated with the loss of protein and mRNA expression. In contrast, the tumours with a single mutation (referred to as ‘one-hit’) exhibited obvious expression levels. Moreover, a mixture of merlin-expressing tumour cells and non-expressing tumour cells was observed in ‘one-hit’ schwannomas, suggesting that a subset of ‘one-hit’ tumour cells was present in these tumours. To mimic the growth promoting effects by the second hit, we performed lentivirus-mediated *NF2* knockdown in the ‘one-hit’ schwannoma cultures. Following the loss of *NF2* expression, schwannoma cultures demonstrated increased proliferation rates. Above all, we have identified a correlation between the *NF2* status and the growth patterns of sporadic VSs. The treatment decision-making, microsurgery or “wait and scan” strategy, should be carried out according to the tumour’s genetic background.

## Introduction

Vestibular schwannomas (VSs) are benign tumours that originate from myelin-forming schwann cells that surround the vestibular branches of the 8th (auditory) cranial nerve. The vast majority (95%) of VSs are unilateral and sporadic schwannomas, which occur predominantly in the 4th to 6th decades of life^1, 2^. In rare cases, they occur as bilateral tumours within the context of the inherited familial cancer syndrome Neurofibromatosis Type 2 (NF2). NF2 is also associated with schwannomas of other cranial, spinal, and cutaneous nerves; meningiomas and, less frequently, ependymomas.

The *NF2* tumour-suppressor gene was first isolated in 1993, when mutations were demonstrated in NF2 patients^3^. The *NF2* gene is located on chromosome 22q 12.2 and encodes a 595-amino acid protein, designated merlin or schwannomin. Alterations in the gene that lead to the development of NF2-related VSs are considered to follow Knudson’s two-hit model^4^, either by mutation in both alleles or by mutation in one allele and the loss of the other allele in conjunction with a loss of heterozygosity (LOH) on chromosome 22^5–7^. Currently, the genetic profiles of sporadic VSs have not been fully characterized. The “two-hits” inactivation of the *NF2* gene that results in total merlin loss has also been demonstrated in sporadic VSs with detection rates ranging from 27% to 61%^7–11^, which is much less than expected. Additionally, conflicting results from merlin expression analyses have been reported in studies on sporadic VSs^11–15^. They are classified by varying protein levels into none (or faint), medium or strong. A possible factor that may impair the sensitivity of these analyses is stromal contamination in the tumour tissues^11^.

The therapeutic management of sporadic VSs mainly includes two strategies: microsurgery or the “wait and scan” strategy. An important aspect in the determination of the most suitable therapy is the tumour growth rate. Some tumours remain stable for years, whereas others grow relatively fast^16, 17^. However, the biological background of these phenotypical heterogeneities is largely unknown. Some studies have reported that fast-growing tumours tend to occur in younger individuals^15, 18^, suggesting there may be a correlation between age at diagnosis and tumour growth rate in sporadic VSs. To understand the genetic events responsible for the clinical behaviours of sporadic VSs, we divide the tumours into three age groups at equally spaced intervals, including 38 young cases (<30 years old), 191 middle-age cases (31–60 years old) and 53 elderly cases (>60 years old), and perform comparisons of the clinical and genetic characteristics between age groups. We are also interested in the implications of protein/mRNA expression and tumour-cell proliferation in response to different *NF2* gene statuses. Therefore, we intend to assess the prognostic impact of the *NF2* gene alterations on tumour growth.

## Materials and Methods

### Ethics statement

All experimental protocols were approved by the Research Ethics Review Committee of Shanghai Jiao Tong University. The methods and experimental protocols in human samples were carried out in accordance with relevant guidelines, and all study participants signed the informed consent form. This study conformed to the provisions of the Declaration of Helsinki.

### Study population

A total of 282 patients with sporadic VSs were included in a multi-center study from March 2008 to September 2015. The patients were divided into three age groups at equally spaced intervals: 38 young cases (less than 30 years old), 191 middle-age cases (between 30 and 60 years old) and 53 elderly cases (more than 60 years old). The two ages (30 and 60 years) were chosen to define age categories because sporadic VSs often occur after 30 years and the incidence rates tend to decrease after 60 years^2^. Five cases of NF2-related VSs were also included as positive controls of genetic analyses. All tumour specimens were resected by the same surgeon, and were then subjected to histological examinations to confirm the diagnosis of schwannomas. Tumour size was measured in terms of the maximal dimension of the lesion on magnetic resonance (MR) imaging. As controls, 10 cases of normal vestibular nerves from vestibular neurectomy for Meniere’s disease were also included. Peripheral blood samples were collected from all patients prior to operation with written informed consent.

### Polymerase Chain Reaction (PCR) and Direct Sequencing Analysis

Bidirectional sequencing was conducted to detect microlesions in the *NF2* gene. DNA extraction from the tumour tissues and blood lymphocytes was performed using the TIANamp Genomic DNA Kit (Tiangen Biotech, Beijing, China), as previously described^19^. The whole coding sequence and the exon–intron boundaries of the gene were amplified by polymerase chain reaction (PCR) using standard methods and underwent bidirectional sequencing as previously described. The sequence data were analysed using Sequencer 4.9 software (Genecode, MI, USA) and compared with the *NF2* sequence (NM_016418) in GenBank. Mutations were described according to the standard nomenclature for DNA sequence changes reported by the Human Genome Variation Society (HVGS).

### Multiplex Ligation-Dependent Probe Amplification Analysis (MLPA)

To identify large/exonic deletions that were not detected by direct sequencing, and to measure the copy number of the *NF2* gene, we used a commercial MLPA kit for the analysis (SALSA P044 NF2; MRC-Holland, Amsterdam, The Netherlands). The *NF2* probemix contains probes for each of the exons of the *NF2* gene. The procedure was based on the manufacturer’s protocol with an initial DNA amount of 75 ng on the 3500 Genetic Analyser (Applied Biosystems, CA, USA). Relative peak heights of all amplicons of each test sample were compared to a normalized average of three vestibular nerves. The results were analysed with MRC-Coffalyser software (MRC-Holland) and the Dosage Quotient (DQ) was used to describe the copy number status. A range of 0.8 < DQ < 1.2 was considered normal, and 0.4 < DQ < 0.7 was considered to show a heterozygous deletion. In addition, three probes located in other regions (*AP1B1*- exon 1, *NIPSNAP1-* exon 10, and *CABP7*- exon 4) on 22q 12.2 were included as positive controls to identify the occurrence of a loss of heterozygosity (LOH) on chromosome 22. Eleven reference probes were included to detect different autosomal chromosomal regions.

### Cell Culture and Transfection

Human Schwann cells (HSCs) were purchased from ScienCell Research Laboratories (catalogue no., 1700; Carlsbad, CA, USA), and cultured in Schwann Cell Medium (ScienCell, Cat. No.1701). For primary schwannoma cell culture, tumour tissues were cut to 2 mm^3^ in size and digested with 0.25% trypsin. The cells were collected and re-suspended in culture medium: DMEM (PAA, Coelbe, Germany), 2.5% Nu-Serum (BD), 0.5 µM forskolin (Sigma) and 10 nM β1-heregulin (Peprotech EC Ltd., UK). To establish merlin-knockdown cultures, short hairpin RNAs (shRNA) were synthesized, annealed, and inserted into a lentivirus vector, containing an independent open reading frame for green fluorescence protein (GFP). Lentiviral shRNA (Lv-shRNA) vectors were constructed using three different shRNA sequences against *NF2*/merlin expression including 5′-ACTTCAAAG ATACTGAC AT-3′ (sh1), 5′-TCTGGATA TTCTGCACAAT-3′ (sh2), and 5′-TTCGTGTTAATAAGCTGAT3′ (sh3). A nonsense shRNA was also constructed using the target sequence 5′-TTCTCCGAACGTGTCACGT-3′ (GeneChem, Shanghai, China). The lentivirus-mediated shRNAs were used to transfect schwannoma cells at a multiplicity of infection (MOI) of 20. To screen the target for the most effective viral transfection, the percentage of GFP-positive cells in the total cell numbers was evaluated under the fluorescence microscope at day 3 following transduction.

### RNA isolation and qRT-PCR analysis

RNA extraction was done using TRIzol reagent according to standard methodology. High-quality RNA was reverse-transcribed into cDNA by a reverse transcription kit (Takara, Dalian, China). PCR amplification was run with SYBR® Premix Ex Taq™ (Takara). Amplification and qPCR measurements were performed on the CFX96 Real-Time PCR Detection System (Bio-Rad). The results were normalized to GAPDH as an endogenous reference (2^−ΔCt^ (*NF2*/*GAPDH*)). The PCR primers were listed as follows: Forward, 5′-GCAGATCAGCTGA AGCAGGA-3′ and reverse, 5′-ACCAATG AGGT TGAAGCTTGGTA-3′ for *NF2*; Forward, 5′AAGGTG A AGGTC GGAG T CAACG 3′ and reverse, 5′ -CAGCCTTCTCCATGGTGGGAA-3′ for *GAPDH*.

### Immunoblot analysis, Immunohistochemistry and Immunofluorescence

The tumour tissues or the collected cells were ultrasonicated in Cell Lysis Buffer (Beyotime, Shanghai China) containing a protease inhibitor (phenylmethanesulfonyl fluoride). Western blotting analysis was performed with antibodies specific for merlin (# HPA003097, Sigma-Aldrich) and cyclinD1 (# C7464, Sigma-Aldrich). The β-actin antibody (#AA128, Beyotime) was used to ensure equal loading of total protein. The protein bands were detected using an chemiluminescence HRP substrate (Millipore). The band densities were quantified using the Imager Lab Software (Bio-Rad). For immunohistochemistry, after deparaffinization and serial ethanol treatment, tumour slices were processed for microwave antigen retrieval pretreatment, digested with 0.3% trypsin, quenched with 3% H2O2, blocked with pre-inoculated goat serum and incubated with anti-merlin antibody (diluted 1:200; # HPA003097) at 4 °C overnight. Biotin-conjugated goat antirabbit IgG secondary antibody and HRP-conjugated avidin were applied at a 1:200 dilution. As a chromogen, 3,3-diaminobenzidine tetrahydrochloride was used, and the sections were counterstained with Haematoxylin. For immunofluorescence, normal cranial nerve samples were processed as in the immunohistochemistry staining (without H2O2 quench). HSCs and schwannoma cells were plated on glass slides, fixed with 4% paraformaldehyde for 20 minutes, and then permeabilized in 0.1% Triton X-100. The tissue slides and the glass slides were blocked with pre-inoculated goat serum before incubation with the anti-S100 (# Z0311, Dako), and anti-merlin (# HPA003097, Sigma). Alexa-488– and Alexa-594–conjugated goat anti-mouse and goat anti-rabbit antibodies were used as secondary antibodies. The Alexa Fluor® 488 Phalloidin (# A12379, Thermo) was used for the staining of the F-actin cytoskeleton. The sections were also nuclear counterstained with 4,6-Diamidino-2-phenylindole (DAPI, Beyotime, China).

### EdU Incorporation Assay and CCK-8 Assays

The 5-ethynyl-20-deoxyuridine (EdU, Ribobio, China) labelling was used to assess the DNA synthesis activity. Briefly, after treatment, the cells were exposed to 50 µL EdU for an additional 24 h at 37 °C, washed with phosphate buffered saline, fixed with 4% formaldehyde for 20 min and incubated with 0.3% Triton X-100 for 20 min. Subsequently, the cultures were reacted with 80 μL of 1 × Apollo® reaction cocktail for 20 min. The DNA contents of the cells in each well were then counterstained with DAPI for 15 min. The effect of merlin knockdown on cell proliferation was determined by a Cell Counting Kit-8 (CCK-8) from Dojindo (Kumamoto, Japan). Briefly, cells were seeded into 96-well plates (4 × 10^3^ cells/well) and grown for 0, 24, 48, or 72 h. At each specified time point, 10 μL/well CCK-8 solution was added and the cells were incubated for an additional 4 h at 37 °C. The absorbance of the wells was measured using a SpectraMax190 microplate reader (Molecular Devices, USA), and the absorbance values (OD values) obtained from three aliquots were averaged to produce a single value to represent cell viability. The absorbance was then expressed in numerical values that were finally subjected to statistical analysis.

### Statistical analysis

The results are expressed as the mean ± SD ranges. Normally distribution and homogeneity of variance tests were performed on the data using SPSS statistics software for Windows (SPSS, Chicago, IL). The statistical analysis of the clinical parameters of the three age groups was performed using the Chi-square test, the one-way ANOVA test, or the Kruskal-Wallis one-way ANOVA. The comparisons of merlin expression between tumours and the cell proliferation index in different treatments were performed using the Student’s t-test or the one-way ANOVA. *P* values of less than 0.05 were considered statistically significant.

### Data Availability

All data generated or analysed during this study are included in this published article (and its Supplementary Information files).

## Results

### Clinical Characteristics

**Figure 1:**
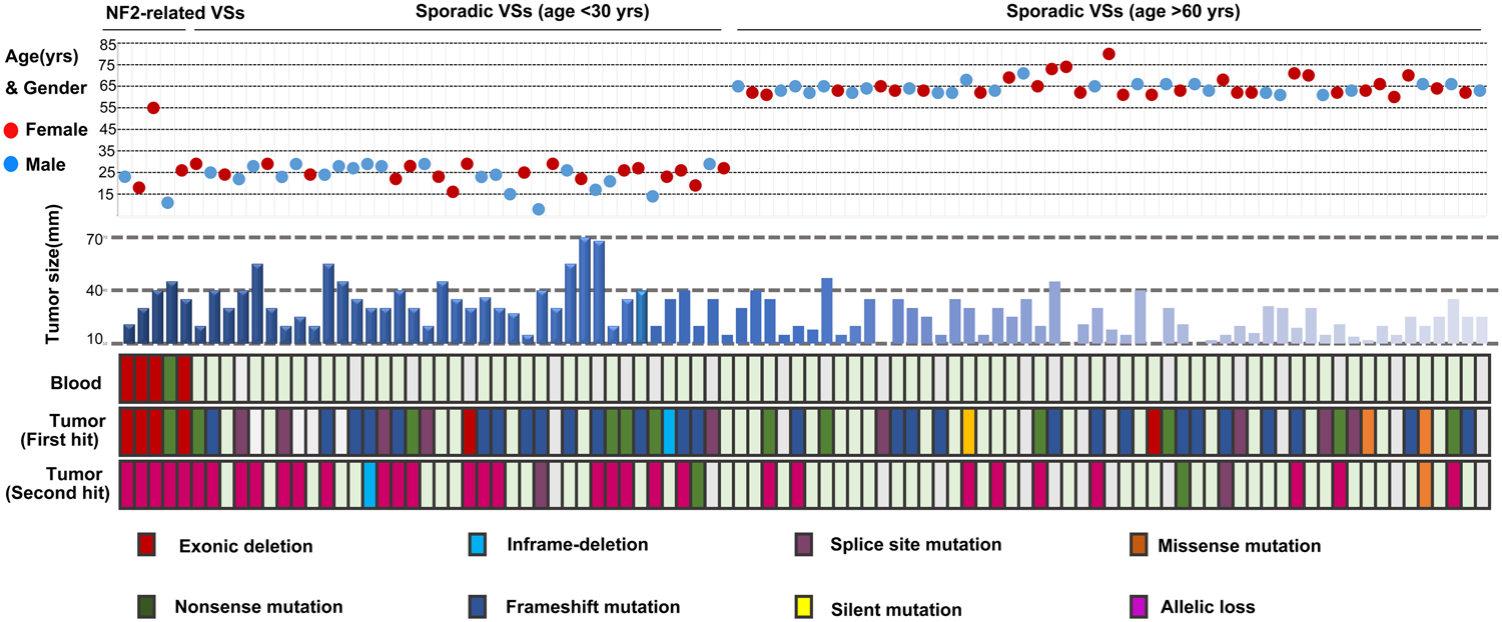
Clinical and genetic characteristics of young and elderly patients with sporadic VSs. The clinical parameters of both young (n = 38) and elderly (n = 53) patients with sporadic VSs, including the age at diagnosis, gender, and tumour size, were present in the upper panel. The screening for the mutation and the copy number of the *NF2* gene was performed using a combination of Direct sequencing and MLPA analysis in these cases. Five NF2-related patients were used as positive controls.

During the study period, 282 patients with sporadic VSs ranging in age at diagnosis from 8 to 85 years (average age = 45.4 years) were identified. For comparison, these individuals were classified in to three age groups: the young group (24.1 ± 4.9 years, n = 38), the middle-age group (47.1 ± 7.9 years, n = 191) and the elderly group (64.7 ± 3.9 years, n = 53). As described in Table 1 (also see in Supplementary Table S1) and Fig. 1, no preference for sex, side or tumour type of the lesion was observed in the three age groups. Interestingly, we found age-dependent differences in the other clinical parameters including the age at the onset of initial symptoms, the duration from the first symptom to diagnosis, and the tumour size. The differences appeared to be more significant between the young group and the elderly group. The young sporadic schwannomas were shown to be characterized by an earlier age for the onset of initial symptoms (mean 22.4 years vs. 60.3 years), a shorter symptom duration (mean 1.6 years vs. 4.4 years; *p* = 0.026) and a larger tumour size (mean 34.3 mm vs. 23.6 mm; *p* = 0.001) compared to the eldery cases. These findings suggested that fast-growing schwannomas tend to occur in young individuals.

**Table 1:** Comparisons of clinical characteristics of sporadic VSs in different age groups.

Clinical characteristics	Young (n = 38)	Middle-age (n = 191)	Elderly (n = 53)	*P* value	Statistical methods
**Age at onset of initial symptom (years)**	22.4 ± 5.4	44.0 ± 8.6	60.3 ± 6.3	0.001	one-way ANOVA
**Gender**					
Male	20	85	25	0.647	Chi-square test Fisher’s Exact test
Female	18	106	28		
**Tumour location**					
Right side	16	102	28	0.438	Chi-square test Fisher’s Exact test
Left side	22	89	25		
**Tumour type**					
Solid	30	135	36	0.110	Chi-square test Fisher’s Exact test
Cystic	8	56	17		
**Size (mm)**	34.3 ± 13.3	26.2 ± 10.2	23.6 ± 9.5	0.001	one-way ANOVA
Stage 2 (1–15 mm)	2	32	15	0.017	Kruskal-Wallis one way ANOVA
Stage 3 (16–30 mm)	17	103	27		
Stage 4 (31–40 mm)	12	42	9		
Stage 5 (>40 mm)	7	14	2		
**Symptom duration (years**)	1.6 ± 2.6	3.3 ± 4.6	4.4 ± 5.3	0.026	one-way ANOVA

### Comparison of the Genetic Characteristics between the Young group and the Elderly group

**Figure 2:**
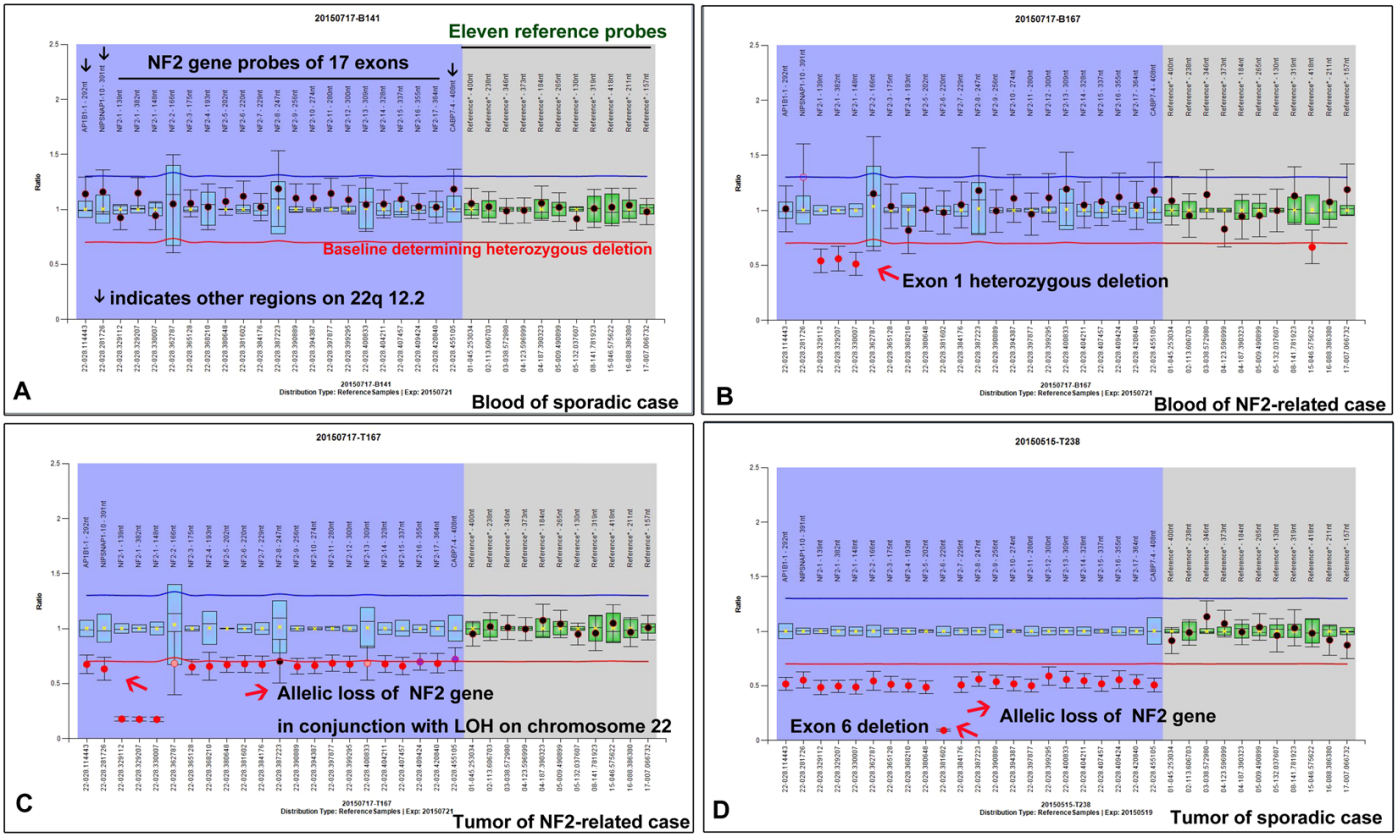
The detection of the exonic deletion and allelic loss of the *NF2* gene by MLPA analysis. **(A)** Three probes located in other regions on 22q 12.2 were included as positive controls to identify a loss of heterozygosity (LOH). Eleven reference probes were included to detect different autosomal chromosomal regions. In the graph, the y-coordinate represented the Dosage Quotient (DQ) and 0.4 < DQ < 0.7 was considered to have a heterozygous deletion. No exonic deletions were found in the blood of sporadic cases. **(B)** In NF2-related VSs, a deletion of exon 1 was seen in the lymphocytes. **(C)** Somatic allelic loss as the second genetic event was demonstrated in matched tumours, in conjunction with LOH. **(D)** Somatic deletion of a single exon (the first hit) followed by the allelic loss of the *NF2* gene were also observed in one case of sporadic VSs.

In an attempt to evaluate the *NF2* mutations relative to tumour behaviours, the detection for the mutation (including exonic deletions) and the copy number of the *NF2* gene was performed using a combination of Direct sequencing and MLPA analysis among the two groups with different clinical characteristics, including 38 cases of young patients and 53 cases of elderly patients with sporadic VSs **(**Fig. 1, also see in Supplementary Table S1 **)**. Five cases of NF2-associated VSs were also included as positive controls for the genetic analyses. The deletion of exon 1 was by far the most common deletion mutation identified in the lymphocytes of NF2 patients^20^. Consistent with the previous finding, four out of the five NF2-related tumours in this study were found to have an exon 1 deletion by Dosage Analysis **(**Fig. 1 and 2B–C **)**. These mutations were demonstrated in their matched blood samples, supporting them as germline mutations. Additionally, positive dosage results were demonstrated in tumours in another three regions on chromosome 22, suggesting that the allelic loss of the *NF2* gene occurred in conjunction with a loss of heterozygosity (LOH) event. However, in sporadic VSs, germ-line mutations were not observed in any blood samples, but somatic mutations were detected in 54 (59.3%) of the 91 cases, including two cases with a deletion of a single exon **(**Fig. 2D **)**. As previously reported^7–9, 15, 19, 21^, most mutations identified were truncating mutations (nonsense, frameshift, and splicing-site mutations) throughout the first 15 exons of the gene. As shown in Table 2, most mutations occurred at the FERM domain of the *NF2* protein (exon 1 to 8), but no particular mutational hot spots were found. Evans *et al*.^22^ reported that the ratio of frameshift to nonsense mutations increased significantly with increasing age at diagnosis in sporadic VSs. However, in our study, the distribution of mutation type did not differ significantly between the two age groups. A second inactivation event was identified in 19 of 26 mutated tumours in the young groups and in 11 of 28 mutated tumours in the elderly groups, including 24 cases with mutation in one allele and a loss of the other, and only 6 cases with two different mutations on each allele. Interestingly, in total, three out (2 young and 1 elderly) of 20 ‘non-mutated’ tumours exhibited allelic loss. Considering these particular cases, the frequency of “two-hits” tumours in the young cases was estimated to be 67.9% (19/28), which was much higher than in the elderly cases (11/29, 37.9%, *p* = 0.046). In contrast, the tumours with a single mutation (here referred to as ‘one-hit’) appeared to occur more frequently than the ‘two-hits’ tumours in elderly patients, with a ratio of 17:11. Therefore, the association of tumour behaviours with the differential *NF2* gene status, i.e., the ‘two-hits’ or the ‘one-hit’, seemed to be established through our comparisons of the clinical and genetic characteristics between the young and elderly patients.

**Table 2:** Comparisons of genetic characteristics between young and elderly sporadic VSs.

Genetic characteristics	Young (n = 38)	Elderly (n = 53)	*P* value
**Mutation rates**	68.4% (26/38)	52.8% (28/53)	0.135
**Mutation types**			
Nonsense mutation	6 (20.7%)	7 (24.1%)	0.965
Frameshift mutation	13 (44.8%)	12 (41.4%)	
Splicing-site mutation	6 (20.7%)	5 (17.2%)	
Others	4 (13.8%)	5 (17.2%)	
**Mutation distributions**			
FERM domain	20 (69.0%)	19 (61.3%)	0.560
α-helical domain	9 (31.0%)	11(35.5%)	
C-terminal domain	0	1 (3.2%)	
**Frequency of ‘two-hits’**	67.9% (19/28)	37.9% (11/29)	0.046

### Merlin Expression in Connection with Detected *NF2* gene Status

**Figure 3:**
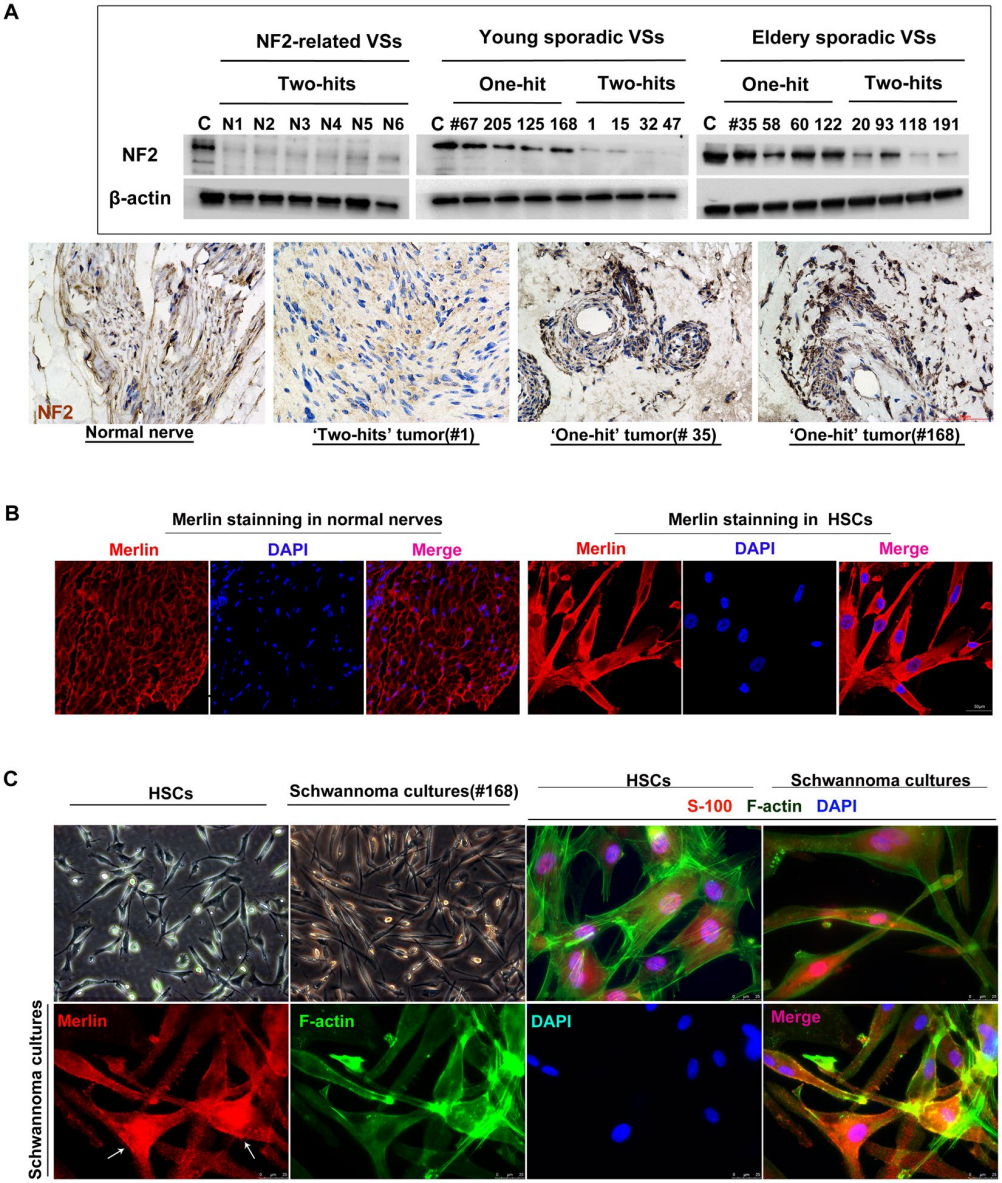
The expression and subcellular localization of merlin protein in sporadic VSs with different NF2 gene statuses. **(A)** The Immunoblot analysis revealed strong merlin levels in the normal vestibular nerve (C, Control). None or very faint 70-kDa merlin-specific bands were demonstrated in six NF2-related schwannomas (N1 to 6) and in eight ‘two-hits’ tumours except for #93. Almost all five ‘one-hit’ tumours were found to exhibit distinct intensity of the 70-kDa band (Upper). A loss of merlin expression (<5% immunostaining) was detected in ‘two-hits’ tumours, while nuclear staining of merlin was observed in ‘one-hit’ tumours (Below). **(B)** The merlin staining of a normal vestibular nerve (left) and HSCs (Right). **(C)** The staining of Schwann cell marker S100 and F-actin in HSCs (Upper left) and schwannoma primary cultures (Upper right). In contrast to the staining homogeneity in HSCs (Upper left), a significant proportion of schwannoma cultures demonstrated strong merlin staining (white arrowheads, Lower panel) and others showed faint staining. Full-length gels are presented in Supplementary Figure 1.

To investigate the association of merlin expression with the detected *NF2* gene status, an antibody recognizing an epitope close to the carboxy-terminus of merlin was used to assess the expression levels of the full-length merlin in schwannomas. The density values of merlin 70-kDa bands were corrected for the difference in the density of actin bands. Protein samples from eight ‘two-hits’ tumours (4 young cases and 4 elderly cases, batch I) and eight ‘one-hit’ tumours (4 young cases and 4 elderly cases, batch II) were prepared. One normal vestibular nerves and six NF2-related schwannomas were also included as the positive and negative controls, respectively. The Immunoblot analysis by anti-actin antibody demonstrated that equal amounts of protein samples were used (Fig. 3A, Lower bands, Upper panel). The strong expression of merlin was detected in the positive control protein extracts of the human normal nerves, as expected (Fig. 3A, Lane 1, Upper panel). In the six negative controls (N1-N6), all of which were NF2-related schwannomas, none or very faint 70-kDa merlin-specific bands were found in short exposure. Similarly, the levels of merlin were drastically reduced in the seven batch I ‘two-hits’ tumours, except for one tumour (#93). The Tumour #93 had a silent mutation (c.114 G > A) in Exon 1 of the *NF2* gene. Reportedly, a silent 2101 A > G transition in exon 9 of the *ATR* gene can cause increased aberrant splicing, either exon skipping of exon 9 or use of cryptic splice donors within exon 9, both of which may introduce a frameshift and stop codon into in exon 10, resulting in low protein levels^23^. It remains unclear whether the silent mutation (c.114 G > A) will influence the transcription, splicing or translation of the *NF2* gene. Because the Tumour #93 showed some expression of full-length merlin, this mutation may serve as a hypomorphic mutation, which might be expected to retain expression of a hypomorphic protein. In contrast, most, if not all, of the batch II tumours were found to exhibit distinct intensity of the 70-kDa bands compared to the bands of batch I, suggesting that the full-length merlin was expressed in batch II tumours, which was consistent with the results from the mutational analyses.

The results from the Immunoblot analysis were further established by immunohistochemistry (Fig. 3A, Lower panel). Loss of merlin expression (<1% immunostaining) was detected in the ‘two-hit’ sporadic VS specimen (#1) and strong staining (>10% immunostaining) was observed in two ‘one-hit’ tumours (#35 and #168). To fully characterized the subcellular localization of merlin in ‘one-hit’ tumours, we performed the fluorescence analysis in schwannoma primary cultures from a ‘one-hit’ tumour (#168), and in human Schwann cells (HSCs). To assure that the cells purified from schwannoma tissues were Schwann cell-derived, the cultures were stained with Schwann cell marker S100 (Fig. 3C, Upper panel). With staining of the F-actin cytoskeleton (green fluorescence; Fig. 3C, Upper right), schwannoma cultures were shown to be predominantly composed of spindle cells, which was consistent with an earlier study^12^. Merlin has been argued to mediate tumour suppression by directly modulating mitogenic signal transduction at or near the plasma membrane, where merlin and the F-actin cytoskeleton were co-localized^24, 25^. The merlin protein was found to be strongly expressed at or near the cell membrane in normal vestibular nerves and HSCs, as expected (Fig. 3B). In contrast to the staining homogeneity in HSCs, approximately 20–30% of schwannoma cells demonstrated obvious merlin staining, but others showed faint staining in short exposure (Fig. 3C, Lower panel). This finding was also demonstrated in Immunohistochemistry on the tumour tissue slides (Fig. 3A, Lower panel). Evans *et al*.^7^ and Elles *et al*.^26^ proposed that the presence of ‘one-hit’ on tumour cells at risk of being transmitted to ‘two-hits’ in sporadic schwannomas should be considered. Therefore, the ‘one-hit’ schwannomas may actually contained both merlin-expressing (hypothesized as the ‘one-hit’) and non-expressing (hypothesized as the ‘two-hits’) tumour cells. As seen in HSCs, the colocalization of merlin and F-actin at the membrane cytoskeleton was shown in schwannoma cultures (Fig. 3C, Lower panel). Interestingly, an obvious proportion of nuclear merlin staining in schwannoma cells was observed and further confirmed in cultures from two additional ‘one-hit’ tumours (data not shown). Nuclear merlin has been suggested to exhibit stronger inhibitory effects than its counterparts at the membrane^27^. The nuclear location of merlin seemed to be associated with the benign behaviours of the elderly tumours, in which the detection rates of ‘one-hit’ were relatively higher than the ‘two-hits’.

### Inactivation of Both *NF2* alleles Suggested Increased Tumour Cell Proliferation

**Figure 4:**
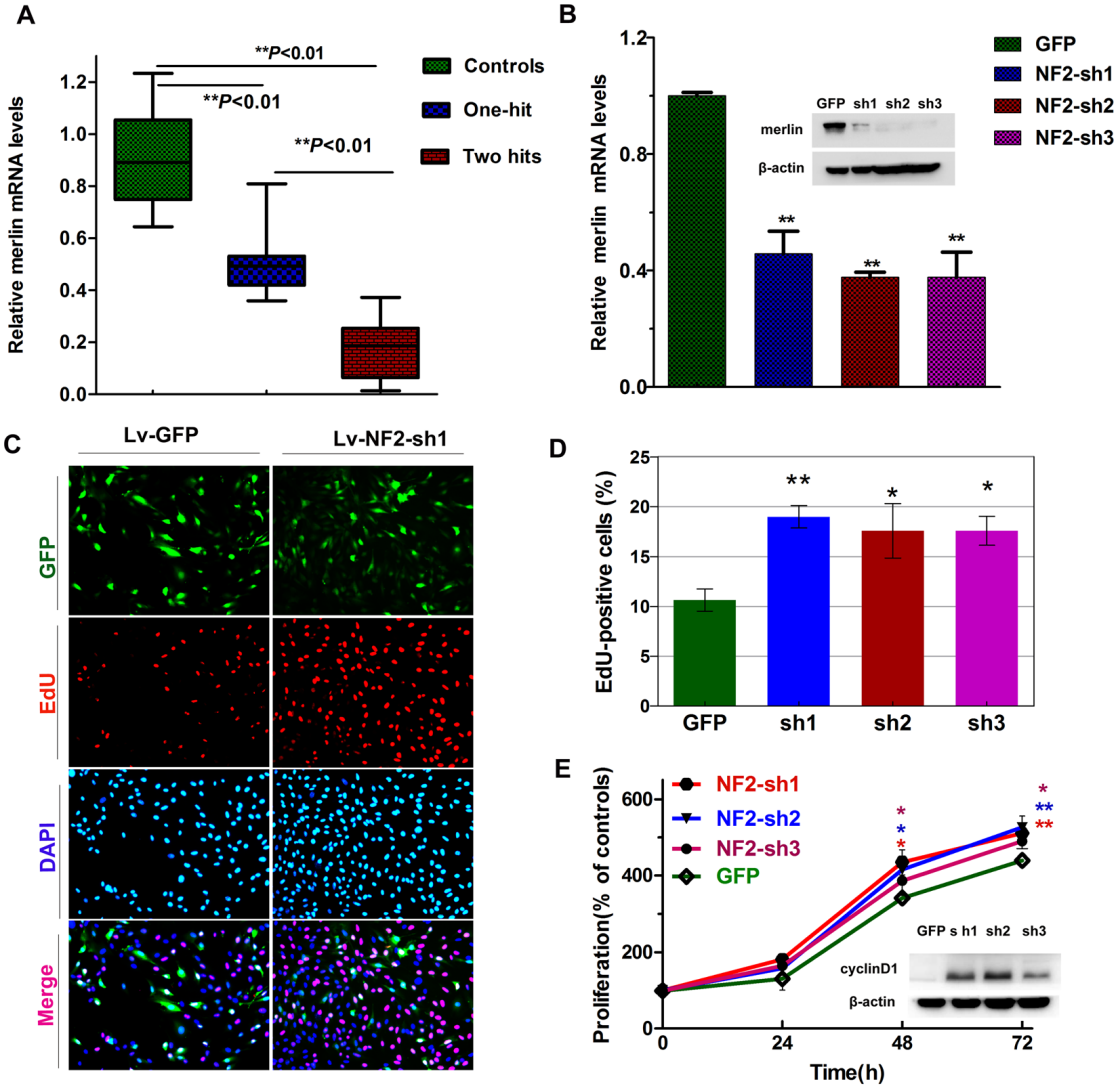
*NF2* gene silencing caused increased tumour cell proliferation. **(A)** The qRT-PCR analysis was carried out to assess the expression level of merlin in 10 cases of ‘two-hits’ tumours, 10 cases of ‘one-hit’ tumours, and 10 vestibular nerves. The details were shown in Supplementary Table S2. (**B**) The expression of merlin in response to *NF2* knockdowns was measured by qRT-PCR and Western Blotting analyses. All qRT-PCR reactions were performed in duplicate. **(C)** Representative images of EdU labelling and DAPI staining were captured under the fluorescence microscope at a magnification of ×200. **(D)** The quantification of EdU incorporating-cells was calculated as follows: EdU-positive cell numbers (red dots)/total numbers (DAPI, blue dots) ×100%. At least three random images were taken from each well. **(E)** The effects of merlin knockdown on cell proliferation were determined by CCK-8 assays. GFP: nonsense shRNA; sh: shRNA. Data were represented as the mean ± SEM (Supplementary Table S3). *p < 0.05 and **p < 0.01. Full-length gels are presented in Supplementary Figure 1.

The mutant *NF2* transcripts are usually underexpressed or inefficiently translated in schwannomas. Not surprisingly, reduced merlin protein levels were observed in ‘one-hit’ tumours. We hypothesized that the expression levels of *NF2* transcripts may be correlated with the number of occurrences of each inactivation hit. To test this notion, qRT-PCR analyses were carried out to assess the expression levels of merlin mRNA in tumours with different *NF2* gene statuses. RNA samples from 10 group I (with single mutations) tumours and 10 group II tumours (including 8 cases with both mutations and 2 cases with mutation plus allelic loss) were included. For the convenience of comparisons, the averaged value obtained for each tumour batch was divided by that in 10 normal vestibular nerves to obtain an indicator ratio. As described in Fig. 4A, significant differences were observed in the merlin expression of nerve/tumour tissues different *NF2* gene statuses (F = 62.263, *p* = 0.001). Merlin mRNA levels remained high in normal vestibular nerves relative to both ‘one-hit’ group I and ‘two-hits’ group II tumours. The statistical analysis revealed that in group II tumours the ratios (0.12 ± 0.17) were much lower than in group I tumours (0.42 ± 0.11, *p* < 0.01), indicating that the second inactivation hit, either mutation or allelic loss, may induce further repressed expression levels of *NF2* transcripts, resulting in the universal loss of merlin expression in the ‘two-hits’ tumours.

Consistent with its role as a tumour suppressor, merlin plays an important role in controlling the proliferation of a variety of cell types^28, 29^. Merlin knockdowns have been demonstrated to cause an increased proliferation rate of human schwann cells (HSCs) *in vitro* ^30^. In contrast, over-expression or reintroduction of the wild-type *NF2* could inhibit the proliferation of HSCs and primary schwannoma cultures by arresting cell cycle at the G0/G1 phase^13, 31^. In the expression analyses, a subset of ‘one-hit’ tumour-cells was suggested in the primary schwannoma cultures from a ‘one-hit’ schwannoma (#168). The presence of merlin expression in ‘one-hit’ schwannomas should be attributed to the existing ‘one-hit’ tumour cells, in which the merlin mRNA was actively expressed by qRT-PCR analysis. To knockdown the merlin expression in these tumour cells, transfection of lentivirus-mediated shRNAs into the schwannoma cell populations was performed. Every experiment was carried out between the second and the fourth passage, when proliferation rates were the best and the number of fibroblasts was negligible (less than 2%, routinely checked by S-100 staining). On day 3 after transfection, each of the knockdown groups and the control groups were harvested and divided into two parts: one part was sent for Western-Blotting/qRT-PCR analyses to verify the presence of efficient merlin silencing and if successful, the remaining part was subjected to an analysis for cell proliferation. As shown in Fig. 4B, the three *NF2* shRNAs (*NF2* sh1, sh2 and sh3) exhibited an excellent extent of reduction in both mRNA and protein expression levels, as compared to the controls. EdU staining was conducted to detect the newly synthesized DNA to determine the proliferation activities. As shown in Fig. 4C and D, since day 3 after seeding the percentage of EdU-positive cells was significantly higher in schwannoma cells with *NF2* shRNAs than in those with nonsense controls. The above results were further confirmed by CCK-8 assays (Fig. 4E), indicating that merlin knockdowns significantly enhanced proliferation rates of schwannoma cell populations. Cyclin D1 is hypothesized to control cell cycle progression through the G1-S check point. The tumour suppressor function of merlin is, at least in part, a consequence of its ability to decrease cyclin D1 expression^13, 14, 32^. Consistent with the phenotypic changes, cyclinD1 overexpression was noted in merlin-knockdown cultures (Fig. 4E). These findings indicated that merlin loss by the second hit on the *NF2* gene induced increased proliferation rates of schwannoma cells. The proportion of ‘two-hits’ tumour cells was also suggested to be the leading factor in the determination of the development (rapid or slow) of schwannomas.

## Discussion

This study investigated the genetic alterations in sporadic VSs, including mutations and allelic loss of the *NF2* gene, and compared these alterations with the clinical characteristics. Sporadic VSs were most commonly seen in the middle-aged individuals with a proportion of 67.8% (191/282), and the tumours in young and elderly individuals were uncommon with proportions of 13.5% (38/282) and 20.6% (53/282), respectively. Here, we found age-dependent differences between the clinical parameters of the three age groups, including the age at the onset of initial symptoms, the symptom duration and the tumour size. The differences were much more significant in the young group than in the elderly group. The progressive clinical behaviours observed in young sporadic VSs led us to explore the underlying genetic mechanisms.

Tumour analysis is more complex than standard lymphocyte DNA analysis. The tumour tissues are not solely composed of tumour cells; they are a heterogeneous collection of both tumour cells and surrounding non-tumourous (or stromal) cells^2, 33, 34^. Schwannomas are prone to contamination by non-tumourous cells such as macrophages, lymphocytes and cells on the blood vessels^11^. Stromal contamination can reduce the detection concentration of tumour cells, and therefore mask the presence of mutation in some samples. This notion may explain the failure to identify a mutation on the *NF2* gene in all of our cases.

The ‘two-hit’ model, which implies a germ-line mutation in one tumour suppressor gene (TSG) allele followed by somatic loss of the remaining wild-type allele, has held true for most TSGs in the explanation of cancer predisposition. The hypothesis has been strongly demonstrated in the *NF2* gene in NF2-related VSs, the familial forms of vestibular schwannomas. The *NF2* gene mutation occurs as the first hit during the development of vestibular schwannomas^5, 7^. In the current study, we found somatic mutations in 54 (59.3%) of the 91 Chinese patients. The results were consistent with the 53–82% detection rates reported in previous studies^7–11^. The allelic loss of the *NF2* gene in conjunction with LOH on chromosome 22 represents the most common form of the second hit in schwannomas^7^. The step-by-step inactivation hits were confirmed by comparisons between mutation patterns of blood and tumour DNA in NF2-related patients. In the genetic study, the mutated sporadic schwannomas however exhibited different *NF2* gene statuses, i.e., the ‘one hit’ and the ‘two-hits’. The presence of only one mutational event in sporadic VSs has also been reported by many studies^7–11^. In young individuals, the ‘two-hits’ schwannomas were present more often than the ‘one-hit’ tumours and the frequency ratio was estimated to be 19:7. In contrast, the ‘one-hit’ appeared to occur more frequently than the ‘two-hits’ in elderly patients, with a ratio of 17:11. The comparison between the genetic profiles of the two age groups with different clinical characteristics may establish a relationship between the detected *NF2* gene status and tumour behaviours. In schwannomas considered as ‘one-hit’ tumours, the methylation of the promoter-associated CpG island may represent the event secondary to the inactivation of the *NF2* gene. Kino *et al*.^35^ reported that the site-specific methylation of the promotor elements was important for *NF2* silencing in sporadic VSs. However, another three studies that included 93 sporadic VSs detected no aberrant methylation of the *NF2* gene in any tumours^8, 36, 37^, suggesting that promoter methylation is an uncommon mechanism of *NF2* inactivation in sporadic VSs.

In expression studies, most of the ‘one-hit’ schwannomas exhibited distinct merlin-bands, but weak or non-bands were observed in all of the ‘two-hits’ tumours, suggesting that the total loss of merlin expression should require the inactivation of both alleles. The ‘one-hit’ *NF2* gene status in schwannomas by our detection tools may not reflect this fact. The development of schwannomas has been reported to be only seen in conditional homozygous *NF2* knockout mice, and not in *NF2* hemizygous mice^6^. Regardless of the stromal cells in schwannoma tissues, it seems unlikely that the so-called ‘one-hit’ tumours contain pure ‘one-hit’ tumour cells. A mutation that causes structural abnormalities in the region of the *NF2* gene may make the locus more susceptible to damage, resulting in the second inactivation hit^38^. It is possible that a subset of ‘one-hit’ tumour cells at risk of being transmitted to ‘two-hits’ may have been present in these schwannomas^7, 26^. The notion was further supported by the merlin staining in the ‘one-hit’ tumour tissues and isolated primary cultures. In contrast to the staining homogeneity in HSCs, the schwannoma cell populations contained a mixture of merlin-stained (hypothesized as ‘one-hit’) and non-stained tumour cells (hypothesized as the ‘two-hits’). The MLPA analysis for the detection of allelic loss may not be sensitive enough to obtain positive results when a high content of ‘one-hit’ tumour cells with a remaining normal allele were present in tumour tissues. The fast-growing schwannomas may have a much larger percentage of ‘two-hits’ tumour cells relative to ‘one-hit’ tumour cells, resulting in positive results in the dosage analyses of tumour DNAs. In other words, the lower percentage of ‘two-hits’ tumour cells in slow-growing tumours may lead to negative results.

To investigate the implications of cell proliferation in response to merlin loss by the occurrence of the second hit, we performed transfection of lentivirus-mediated *NF2*-shRNAs into the schwannoma cultures to knockdown merlin expression. Following the loss of merlin expression, schwannoma cultures demonstrated increased proliferation rates, as expected. The finding suggested that ‘two-hits’ tumour cells exhibited a stronger proliferation activity than the ‘one-hit’ tumour cells. Therefore, it was not surprising that higher detection rates of the ‘two-hits’ were observed in young schwannomas characterized by progressive behaviours. The finding also suggested that the ‘two-hits’ tumour cells may play a leading role in the clinical progression of sporadic schwannomas.

According to current studies, we postulated that the detected *NF2* gene status was correlated with the clinical behaviors of sporadic VSs. The ‘one-hit’ schwannomas, probably containing a high content of ‘one-hit’ tumour cells, may exhibit slower growth rates compared to their ‘two-hits’ counterparts. Therefore, the treatment decision-making, the microsurgery or the “wait and scan” strategy, should be chosen according to genetic backgrounds. The cerebral spinal fluid (CSF) circulates through the CNS and has a large interface with the vestibular schwannoma tissues. Recent studies have demonstrated the great potential of tumour-derived cell-free DNA (cfDNA) from CSF for cancer prognosis^39–41^. Future improvements of the non-invasive detection techniques concerning CSF cfDNA may provide detailed genetic information on patients with sporadic VSs, especially the proportions of ‘one-hit’/‘two-hits’ tumour DNA, generating a prognostic impact on the clinical treatment strategies.

## Electronic supplementary material

Supplementary information

Supplementary Table S1

Supplementary Table S2

Supplementary Table S3
