# Supplementary material for: Differential NF2 Gene Status in Sporadic Vestibular Schwannomas and its Prognostic Impact on Tumour Growth Patterns
Source: Sci Rep. 2017 Jul 14;7:5470. doi: 10.1038/s41598-017-05769-0 (PMC5511254; doi:10.1038/s41598-017-05769-0)
Supplement: Supplementary file 1 — Supplementary information [file 41598_2017_5769_MOESM1_ESM.pdf]

Supplementary information

Differential NF2 Gene Status in Sporadic Vestibular Schwannomas and its Prognostic Impact on Tumour Growth Patterns

Hongsai Chen [1, 2, 3, 4 #], Xue Lu[1, 2, 3, 4#], Hantao Wang [1, 2, 3, 4#], Zhaoyan Wang [1, 3, 4*] and Hao Wu [1, 3, 4*]

Institutional affiliation:

1. Department of Otolaryngology Head & Neck Surgery, The Ninth People's Hospital, Shanghai Jiao Tong University School of Medicine, Shanghai, China
2. Department of Otolaryngology Head and Neck Surgery, Xinhua Hospital, Shanghai Jiao Tong University School of Medicine, Shanghai, China
3. Ear Institute, School of Medicine, Shanghai Jiao Tong University, Shanghai, China
4. Shanghai Key Laboratory of Translational Medicine on Ear and Nose Diseases, Shanghai, China

---

[#] **Indicated authors contributed equally to this work**

***Correspondence to: Zhaoyan Wang (wzyent@126.com ✉)**

& Hao Wu (wuhao622@sina.cn ✉)

Telephone number: 86-021-25078891 Fax number: 86-021-65156489

Address: Department of Otolaryngology Head & Neck Surgery, The Ninth People's Hospital, School of Medicine, Shanghai Jiao Tong University, No. 639, Zhi-Zao-Ju Road, Shanghai 200011, China

Competing interests

The authors disclose no potential conflicts of interest.

The Supplementary information included Supplementary Figure 1.

Supplementary Figure 1

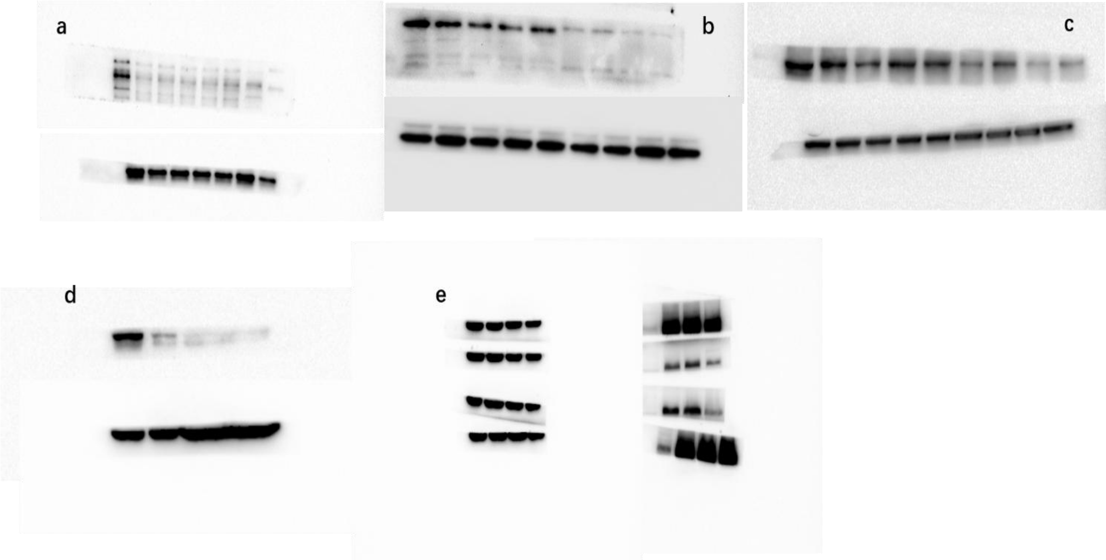

Figure s1. Full-length gels in Western Blotting analyses. (a-c) for Fig 3A： merlin expression in correlation with NF2 gene status. **(d)** for Fig 4B：changes of merlin(upper) expression in response to NF2 knockdowns. The actin bands were presented in the lower panels. **(e)** for Fig 4E: changes of cyclinD1(left) expression in response to NF2 knockdowns. The actin bands were shown in the right panel. Four repeated gels were presented.
